# Vibrio Phage Artemius, a Novel Phage Infecting *Vibrio alginolyticus*

**DOI:** 10.3390/pathogens11080848

**Published:** 2022-07-28

**Authors:** Stavros Droubogiannis, Lydia Pavlidi, Maria Ioanna Tsertou, Constantina Kokkari, Dimitrios Skliros, Emmanouil Flemetakis, Pantelis Katharios

**Affiliations:** 1 Hellenic Centre for Marine Research, Institute of Marine Biology, Biotechnology & Aquaculture, 71500 Heraklion, Greece; stavros.drou@gmail.com (S.D.); lydiapav@gmail.com (L.P.); tsertou@hcmr.gr (M.I.T.); dkok@hcmr.gr (C.K.); 2 Department of Biology, School of Sciences and Engineering, University of Crete, 71500 Heraklion, Greece; 3 Department of Biotechnology, Agricultural University of Athens, 11855 Athens, Greece; dsklhros@gmail.com (D.S.); mflem@aua.gr (E.F.)

**Keywords:** *Siphoviridae*, phage therapy, aquaculture, *Vibrio alginolyticus*

## Abstract

*Vibrio alginolyticus* is an important pathogen of marine animals and has been the target of phage therapy applications in marine aquaculture for many years. Here, we report the isolation and partial characterization of a novel species of the *Siphoviridae* family, the Vibrio phage Artemius. The novel phage was species-specific and could only infect strains of *V. alginolyticus*. It could efficiently reduce the growth of the host bacterium at various multiplicities of infection as assessed by an in vitro lysis assay. It had a genome length of 43,349 base pairs. The complete genome has double-stranded DNA with a G + C content of 43.61%. In total, 57 ORFs were identified, of which 19 were assigned a predicted function. A genomic analysis indicated that Vibrio phage Artemius is lytic and does not harbor genes encoding toxins and antibiotic resistance determinants.

## 1. Introduction

*Vibrio alginolyticus* is one of the most important pathogenic bacteria of marine organisms and has become a serious threat to the aquaculture industry [1]. It has been reported to be a causative agent of vibriosis in fish [2,3], crustaceans [4,5] and mollusks [6] all over the world. The pathogen is particularly relevant to the Chinese aquaculture industry where it has been associated with severe economic damage [7]. In European marine aquaculture, it is a common inhabitant of fish and bivalve hatcheries and it has been linked to larval mortality when poor water quality favors its growth, mostly in live feed departments [8]. The management of bacterial infections in aquaculture is mainly conducted with antibiotics, a practice that is currently under close scrutiny because of the risk of the development of antimicrobial resistance and its effect on the environment and consumers. In addition, there are cases where the use of antibiotics or disinfectants is not advisable or cannot be considered at all. An aquaculture hatchery, for example, is a delicate environment where bacterial communities are in a dynamic balance and play a crucial role in the survival, development and performance of the developing fish or bivalve larvae [9]. Therefore, the destruction of this microbial balance may lead to dysbiosis with detrimental consequences [10]. In this regard, phage therapy may offer a unique advantage in the modulation of the microbial population because phages are highly host-specific and can selectively target pathogenic bacteria without affecting the beneficial ones. Phage therapy has become very attractive in aquaculture; this is evident from the increasing research efforts observed in the past years [11,12,13]. 

Here, we describe the isolation and characterization of a novel phage infecting *Vibrio alginolyticus* isolated from the live feeds of a fish hatchery. This work is part of a broader effort of our research team to isolate and characterize lytic bacteriophages against pathogenic vibrios relevant to marine aquaculture [8,14,15,16] toward the creation of potent phage cocktails that can be used as an alternative treatment for vibriosis.

## 2. Materials and Methods

### 2.1. Bacterial Strains 

The bacterial strain used as a host (HCMR-2 Art1) was isolated from the water of a live feed *Artemia salina* culture tank of the Institute of Marine Biology, Biotechnology and Aquaculture of the Hellenic Centre for Marine Research in Heraklion, Crete (IMBBC-HCMR). HCMR-2 Art1 was identified as *Vibrio alginolyticus* using molecular methods (PCR amplifying 16S rRNA, MreB, toxR) [17,18,19] and whole genome sequencing. In addition to the original host, several clinical and environmental strains of the same species as well as other species of the genus from HCMR’s collection were used in order to assess the host range of the phage (**Table 1**). All bacterial strains were maintained in microbeads (MicroBank) at −80 °C; when working, they were grown in a lysogeny broth (LB) (23.4 gL^−1^ NaCl, 24.7 gL^−1^ MgSO_4_ × 7H_2_O, 1.5 gL^−1^ KCl, 1.43 gL^−1^ CaCl_2_ × 2H_2_O, 1% tryptone and 0.5% yeast extract) at 25 °C. All strains had been previously identified to a species level using sequencing.

### 2.2. Phage Isolation

The phage was isolated from the water of a live feed *Artemia salina* culture tank of IMBBC-HCMR following a standard enrichment procedure [20]. Briefly, 1 L of the sample water was enriched with 100 mL of 10 × LB medium, inoculated with 10 mL of an overnight culture of the host bacterium (*Vibrio alginolyticus* strain HCMR-2 Art1) and incubated at 25 °C for 24 h. Following filtration through 0.22 µm filters, 10 µL aliquots were plated by a standard double-layer agar method and incubated overnight at 25 °C to detect and enumerate the plaque forming units (PFU). The clearest plaque formed was picked and further purified by re-plating five times to ensure clonal phage stocks. The purified phage was named Vibrio phage Artemius and propagated to reach a titer of 10^10^ PFU mL^−1^ and stored at 4 °C.

### 2.3. Phage Morphology

The phage morphology was observed by transmission electron microscopy (TEM). An aliquot of the phage suspension with a titer of ~10^10^ PFU mL^−1^ was negatively stained with 4% *w*/*v* uranyl acetate. Vibrio phage Artemius was observed using a JOEL transmission electron microscope operated at 60 kV at the Electron Microscopy Lab of the University of Crete.

### 2.4. Host Range

The host range of Vibrio phage Artemius was evaluated by a spot test using the bacterial stains shown in Table 1. A total of 1 mL of fresh culture of each bacterial strain (10^7^ CFU mL^−1^) was mixed with 3 mL of warm top agar (culture medium with 0.6% agar) and poured onto bottom LB 1/2 agar plates. When the top agar hardened, spots of 10 µL phage were made and examined after 24 h incubation for lysis. 

### 2.5. In Vitro Efficacy

The efficacy of Vibrio phage Artemius on the host strain was assessed by an in vitro lysis assay. Sterile 96-well plates were used and loaded with 180 μL of a freshly prepared culture of the host bacterium. The plate was placed in a TECAN microplate reader (Infinite PRO 200) equipped with a temperature control and incubated at 25 °C with orbital shaking. When the bacterial culture was at the exponential phase (~10^7^ CFU mL^−1^), it was infected with 20 μL of the phage preparation at 4 different MOIs (0.1, 1, 10 and 100 in triplicate). Three wells were not infected and were used as the control. The growth curve of the cultures was monitored in real-time over a minimum of 18 h and OD600 measurements were recorded every 10 min.

### 2.6. Phage Stability at Different Temperatures and pH

The thermal stability of the novel phage was examined by exposing 500 μL aliquots of the phage (titer: 10^7^ PFU mL^−1^) to different temperatures (4, 25, 35, 45, 65, 70 and 80 °C). The aliquots were incubated at each temperature for 1 h and then rested at room temperature (RT) for 10 min. Each aliquot was then serially diluted and spotted (10 μL/spot) on a host bacterial lawn (HCMR-2 Art1). After a 24 h incubation of the agar plates, the phage titer was determined for each temperature and 4 °C was used as the control. 

The sensitivity of the phage in acidic and alkaline environments was determined by exposing the phage to different pH values according to Pan et al. [21]. A scale of pH ranging from 1 to 10 was made by adding NaOH or HCl to the LB broth agar. The phage was suspended at each different pH value with a final titer of 10^7^ PFU mL^−1^ and stored at 4 °C. After 2 h, and then 10 min of rest at RT, the aliquots were serially diluted and spotted onto the host bacterial lawn. After a 24 h incubation of the agar plates, the phage titer was determined for each pH value and pH = 7 was used as the control. Both the thermal and pH stability was tested in triplicate. 

### 2.7. Adsorption Time and One-Step Growth

For the adsorption time and one-step growth, we followed the protocol described in Misol et al. [16] according to Clokie et al. [22] with a few modifications in triplicate. For the adsorption time, a fresh host culture at the exponential phase (~10^7^ CFU mL^−1^) was infected with the phage at a multiplicity of infection (MOI) of 0.01. The same amount of phage was added to a tube containing only LB and served as the control. Immediately after the infection, 500 μL of the infected culture was placed in tubes containing chloroform and stored in ice (4 °C). The same procedure was repeated for 20 min with a 2 min interval. When all time points were collected, the aliquots were centrifuged at 13,000 rpm for 3 min. They were then serially diluted and spotted onto the host bacterial lawn on LB ½ agar plates. The phage titer was determined after 24 h of incubation of the agar plates at 25 °C.

For the one-step growth, 1 mL of freshly grown culture at the exponential phase (~10^7^ CFU mL^−1^) was centrifuged at 13,000 rpm for 3 min. The supernatant was discarded and the pellet was washed with saline. After two repetitions of this step, the culture was finally resuspended in LB, infected with the phage at a MOI of 0.01 and rested for 15 min at RT. Following centrifugation at 13,000 rpm for 3 min, the supernatant was discarded and the pellet was dissolved in 1 mL saline. The infected culture was then transferred to a new tube containing 25 mL LB and the aliquots were immediately removed and placed in empty Eppendorf vials. The aliquots were then centrifuged at 13,000 rpm for 3 min and the supernatant was transferred to a new 2 mL Eppendorf vial, serially diluted and spotted onto the host bacterial lawn on LB ½ agar plates. This procedure was repeated every 10 min for 120 min. The phage titer was determined after 24 h of incubation of the agar plates at 25 °C.

### 2.8. Genomic Analysis

The DNA extraction of Vibrio phage Artemius was performed using the phenol–chloroform method according to Higuera et al. [20]. The extracted DNA was visualized for quality via 1% agarose gel electrophoresis at 80 kV for 1 h with a 50 kbp ladder. Milli-Q^®^ Reference Water (Merck KGaA, Darmstadt, Germany) was used as a negative control. At least 5 μg of high-purity bacterial DNA, the quality of which was tested using a BioAnalyzer (Bio-Rad, Chicago, CA, USA), was used to generate a paired-end 300 PE genomic library with an optimized size selection using magnetic bead purifications based on the standard Illumina protocol and by using a Nextera XT Library Construction Kit (Illumina, San Diego, CA, USA). To calculate the average size of the library, we used a Tapestastion 4200 system (Agilent, New York, CA, USA). The insert size was estimated as the average size of the library minus the Illumina adapter size and was found to be 834 bp. The sequencing was performed using an Illumina NovaSeq 6000 sequencing platform (Illumina, San Diego, CA, USA) according to the manufacturer’s protocol at Life Sequencing (Valencia, Spain), which allowed us to obtain at least 1 million paired reads sequences. Possibly contaminated, primer, N-terminus and 3′-, 5′-low quality reads were trimmed off (threshold: 0.05). The raw reads were quality inspected and were assembled in Geneious Prime using the Geneious assembler. RASTk and Glimmer were used as the gene predictors for the structural annotation through the PATRIC webserver [23] and Galaxy webserver [24]. The presence of a phage start codon (ATG/GTG or TTG) was manually validated and a Shine–Dalgarno feature was added to all features that had a detectable match. The functional annotation of the predicted proteins was conducted manually using the NCBI Basic Local Alignment Search Tool (BLAST) [25] adjusted at a non-redundant (nr) protein database as well as the Gene Ontology [26], InterPro [27] and TΜHMM 2.0 [28,29] databases. The NCBI Conserved Domain Database (NCBI CDD) [30] was used to detect the conserved regions within the predicted proteins. Genes associated with integration, virulence and antibiotic resistance in the phage genome were searched for using the INTEGRALL Database webserver [31] and Virulence Factor Database (VFDB) [32] as well as the VirulenceFinder and ResFinder webservers [33]. The phage genome was scanned for the presence of tRNAs using the ARAGORN tool [34] through the Galaxy server [24]. The phage lifestyle was also assessed using the Phage AI platform (PhageAI S.A. Artificial Intelligence & Bioinformatics for Phage Research, https://phage.ai/, accessed on 1 May 2022). The genome of Vibrio phage Artemius with annotated predicted ORFs was then visualized in a circular representation with Geneious software (Geneious v9.1, Biomatters, Auckland, New Zealand, http://www.geneious.com, accessed on 1 May 2022) and CGview.

### 2.9. Phylogeny

A ViPTree was used to build a viral proteomic tree by comparing the proteome of Vibrio phage Artemius with 4982 dsDNA phage proteomes [35]. The phylogenetic relationship between Artemius and other similar Vibrio phages was determined using Molecular Evolutionary Genetics Analysis (MEGA X) software (**Table 2**) [36]. A total of 14 large terminase subunits of the described Vibrio phages were downloaded from the NCBI VIRUS database and were aligned with the large terminase subunit of Vibrio phage Artemius using the MUSCLE algorithm [37]. The gaps in the amino acid sequence alignments were trimmed. A maximum likelihood phylogenetic tree was constructed using the TN93 model [38] with a bootstrap test = 1000. The Interactive Tree of Life webserver [39] was used to visualize the constructed tree.

## 3. Results

**Figure 1:**
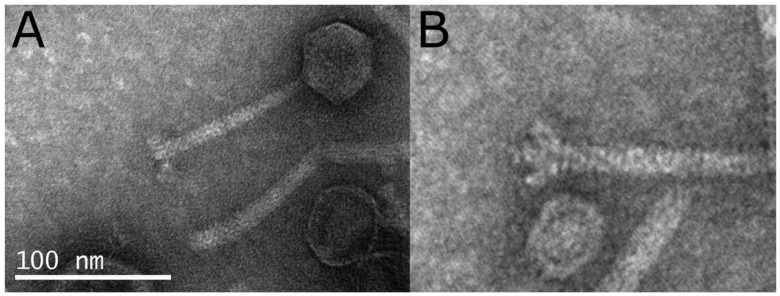
(**A**) Transmission electron micrograph of Vibrio phage Artemius. (**B**) Details of the baseplate structure at the distal end of the tail.

The *phage morphology* of the virions as observed with TEM classified the novel phage to the *Siphoviridae* family. The head was 48.7 ± 0.9 nm wide and the tail was 107.0 ± 2.9 nm long (average ± S.E, n = 15) (**Figure 1**).

### 3.1. In Vitro Efficacy

**Figure 2:**
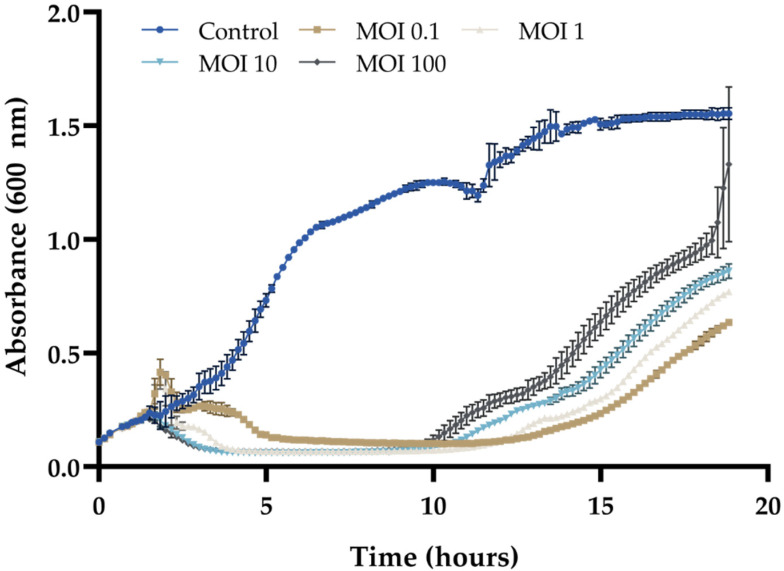
In vitro cell lysis of Vibrio phage Artemius vs. host strain HCMR-2 Art1. The conditions tested were a control (HCMR-2 Art1 strain without any phage addition) and 4 different MOIs used for infecting HCMR-2 Art1 (MOI = 0.1, MOI = 1, MOI = 10 and MOI = 100). The values are means ± standard deviations of the three replicates.

Vibrio phage Artemius was able to lyse the host bacterial population in vitro using approximate MOIs ranging between 0.1 and 100 (**Figure 2**), with the highest MOI yielding a more rapid decrease in the bacterial titer. The bacterial titer remained low (close to the detection limit) over a period of approximately 10 h.

### 3.2. Host Range

According to the host range assay, Vibrio phage Artemius was able to only lyse strains that belonged to *V. alginolyticus* and none of the other bacteria that were tested in the study (**Table 3**). 

### 3.3. Thermal and pH Stability 

**Figure 3:**
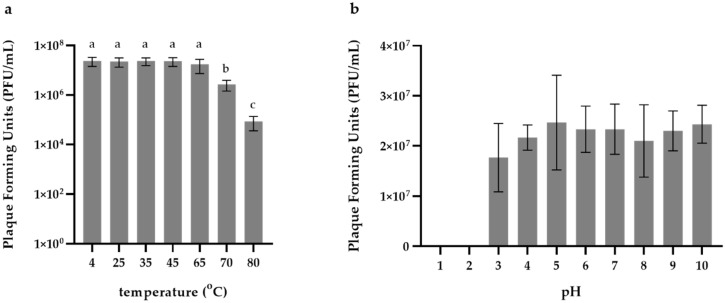
**Thermal and pH stability.** Vibrio phage Artemius exposed to (**a**) different temperatures, with T = 4 °C used as the control, and (**b**) different pH values, with pH = 7 used as the control. Error bars represent the standard deviation of the mean (n = 3). Statistical significance is indicated by different letters above the bars; *p* < 0.05.

We exposed Artemius to different temperatures ranging from 25 °C to 80 °C, with T = 4 °C serving as the control (Figure 3a), to investigate its thermal stability. We found that Artemius was stable up to 65 °C with no significant difference in the titer compared with the control. A reduction of the titer (one-way ANOVA, *p* < 0.05) was observed when the phage was exposed to 70 °C and 80 °C. The phage was not inactivated at high temperatures. We then exposed the phage to acidic and alkaline pH values ranging from pH = 1 to pH = 10 and compared the titer of the phage with the titer at pH = 7, which served as the control (Figure 3b). The phage was inactivated when exposed to pH = 1 and pH = 2 (one-way ANOVA, *p* < 0.001), but remained stable from pH = 3 to pH = 10 with no statistically significant difference compared with the control. 

### 3.4. Adsorption Time and One-Step Growth

**Figure 4:**
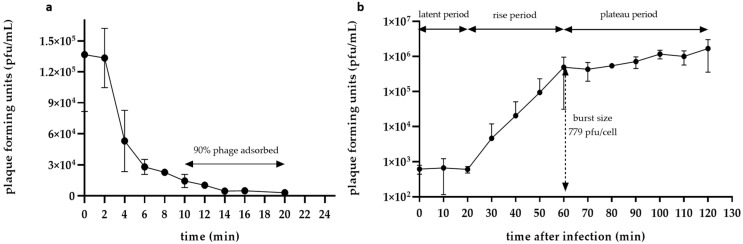
(**a**) Adsorption time assay: at 10 min, 90% of phage Artemius was irreversibly adsorbed by the host bacteria. (**b**) One-step growth curve: latency period for Artemius was 20 min and the burst size was 779 PFU cell^−1^. Error bars represent the standard deviation of the mean (n = 3).

The adsorption time assay showed that 90% of the Vibrio phage Artemius virions required 10 min to bind to the bacterial host (Figure 4a). Artemius had a latent period of 20 min and a rise period between 20 and 60 min. The plateau phase, when no more phages were released from the host cell, was reached at 60 min. The burst size was calculated to be 779 PFU cell^−1^.

### 3.5. Genomic Analysis

**Figure 5:**
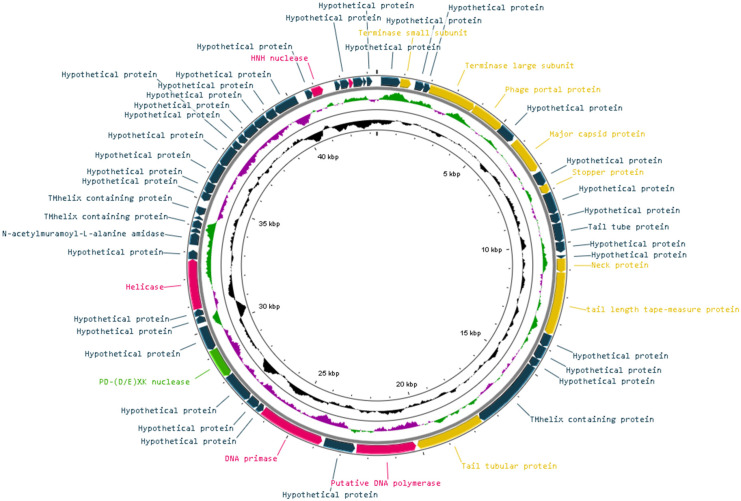
Visual representation of the Vibrio phage Artemius genome in which the genome GC content is shown by the inner black line and the GC skew guanine over-representation by the inner purple/green line. The predicted ORFs are shown as arrows. The color of the ORFs refers to the annotated biochemical function: phage assembly proteins (brown); DNA replication, repair and recombination associated proteins (purple); auxiliary metabolic proteins (light green); and hypothetical (dark green).

The genome size of Vibrio phage Artemius was 43,349 bp with a GC content of 43.61%. The genome arrangement was dense, as suggested by the 1.31 genes per kbp. The Rapid Annotation using Subsystem Technology (RASTk) server and Glimmer.hmm 3.0 revealed that 57 ORFs were present in the genome. Each individual ORF was manually inspected in order to validate the presence of start codons (ATG/GTG or TTG). There was no presence of tRNA in the genome, as shown by ARAGORN. A total of 50 ORFs used a start codon of ATG, 6 ORFs used GTG and 1 used TTG. A search of the NCBI nr database showed that 45 ORFs (78.95%) had significant hits (expected value ≤ 10^−3^) with an average similarity of 52.67%. Overall, 19 ORFs (10.83%) were assigned a function based on the protein homology. No genes associated with integration, virulence or antibiotic resistance were detected. The genome of Vibrio phage Artemius was not modularly organized (**Figure 5**). However, several gene encoding proteins required for phage assembly (ORF 2, ORF 5, ORF 6, ORF 8, ORF 10, ORF 13, ORF 16, ORF 17 and ORF 22) were arranged in subclusters as well as a few genes encoding for DNA replication and nucleotide metabolism proteins (ORF 25, ORF 33, ORF 51 and ORF 54). The genes that were functionally annotated are shown in **Table 4**.

**Figure 6:**
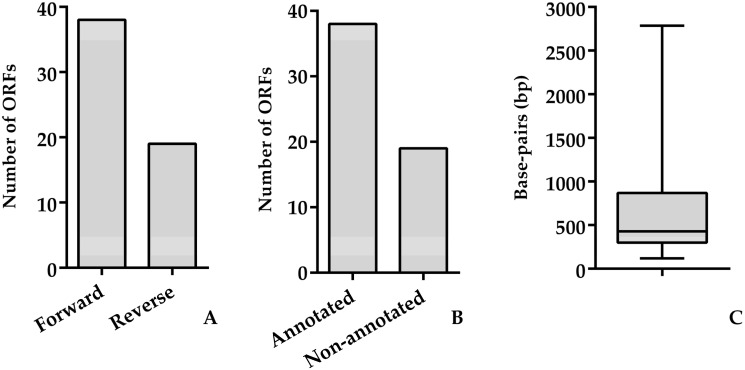
(**A**) Number of ORFs in a forward and reverse direction. (**B**) Number of annotated and non-annotated ORFs. (**C**) Length of ORFs.

The proteins required for phage morphogenesis included major capsid protein (ORF 8), tail-length tape measure protein (ORF 17), tail tube protein (ORF 13), tail tubular protein (ORF 22) and neck protein (ORF 16). In addition, the small terminase subunit (ORF 2), large terminase subunit (ORF 5), stopper protein (ORF 10) and phage portal protein (ORF 6), which are involved in DNA packaging for tailed phages, were identified. Proteins for DNA replication, recombination and repair were also detected: these were DNA polymerase (ORF 23), HNH endonuclease (ORF 51), DNA polymerases, DNA helicase (ORF 33, DNA primase (ORF 25) and other regulatory elements (ORF 54). Finally, several transmembrane proteins were detected (ORF 21, 35, 37 and 39) (**Figure 6**). 

### 3.6. Phylogenetic Analysis

**Figure 7:**
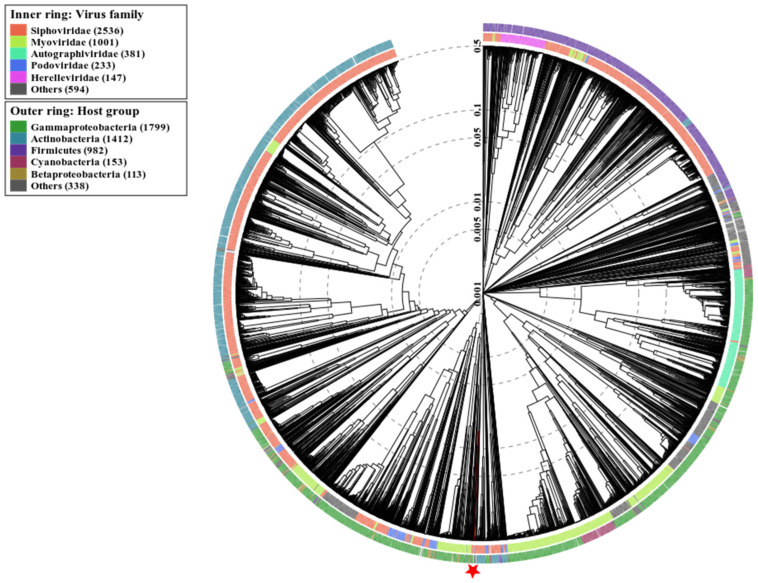
Prediction of taxa and host groups for Vibrio phage Artemius according to the proteomic tree produced by ViPTree. The novel phage was determined to belong to the *Siphoviridae* family and infect the Gammaproteobacteria group (red star and line). The Vibrio phage Artemius (asterisk) proteome was compared with 4892 dsDNA phage proteomes. The tree was rooted using “midpoint rooting”. The branch length scale was calculated as log values. The inner and outer ring indicate the taxonomic virus family and host group, respectively.

**Figure 8:**
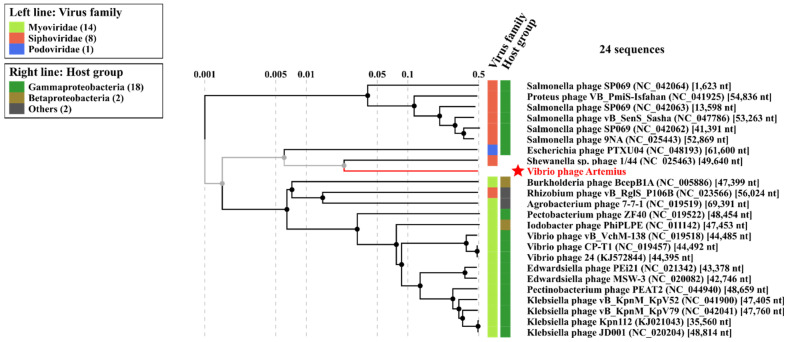
A closer view of the proteomic tree analysis by ViPTree focusing on the closer-related phages of Vibrio phage Artemius.

A wide genome proteomic tree analysis validated that Vibrio phage Artemius belonged to the *Siphoviridae* taxonomic family (**Figure 7**). In addition, it was predicted to infect hosts from the Gammaproteobacteria class, which includes the *Vibrionaceae* family. A closer view of the tree focusing on the nearest relatives (**Figure 8**) showed that Vibrio phage Artemius clustered together with Shewanella phage 1/44 (*Siphoviridae*) and Escherichia phage PTXU04 (*Podoviridae*).

**Figure 9:**
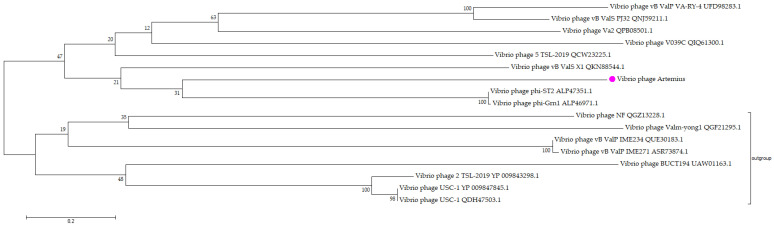
Phylogenetic tree of Vibrio phage Artemius with other *Vibrio alginolyticus* phages. The nucleotide sequences of large terminase subunits of phages were downloaded from the NCBI database and aligned using MUSCLE and a maximum likelihood (bootstrap = 1000). A rooted phylogenetic tree was constructed using MEGA X. Gene sequences with less than 20% similarity with the obtained sequence were arbitrarily set as an outgroup. The bootstrap support value is denoted in each branch as a percentage.

Phylogeny using the large terminase subunits of the *Vibrio alginolyticus* phages (**Figure 9**) showed that Vibrio phage Artemius shared a common ancestor with Vibrio phage phi-St2 and Vibrio phage phi-Grn1. However, the phages were rather distant, resulting in low bootstrap values (<75%); thus, the node was not well-supported. A large terminase subunit gene was used for the phylogeny because it is considered to be a signature, well-conserved gene among the phages and is a strong molecular motor associated with phage packaging [40].

## 4. Discussion

*Vibrio alginolyticus* has been used frequently as a target for novel phage isolation. Until today, more than 40 different phages have been isolated against *V. alginolyticus*, which is suggestive of the importance of this opportunistic pathogen for the aquaculture industry [41]. Several reviews have been published on the topic of phage therapy in aquaculture and the existing challenges [11,42], but also specifically on the interaction of phages with vibrios [13].

Vibrio phage Artemius is a novel phage, as indicated by the BLAST search in the NCBI nr database where the closest phage at the nucleotide level was Vibrio phage 2.044.O._10N.261.51.B8 (MG592661.1) with a 79.6% similarity over a 2% query cover. A phylogenetic analysis of Artemius verified the novelty of this phage. According to the phylogeny obtained with the terminase large subunit using phages infecting the same host (*V. alginolyticus*), Vibrio phage Artemius was closer to Vibrio phage phi-St2 and Vibrio phage phi-Grn1, which belong to the genus of *Schizotequatrovirus* [43]. According to the ViPTree analysis, which uses the whole proteome of all described phages, Vibrio phage Artemius was closer to siphovirus 1/44 infecting *Shewanella* sp. Interestingly, this phage was isolated from iced water in the Baltic Sea [44]. The diversity of the phages and the mosaicism of their genomic arrangement make a phylogenetic analysis using traditional phylogenetic trees difficult and occasionally vague, especially when phage species are under-represented [45,46]. 

Two of the most crucial characteristics of a phage to be considered for therapeutic purposes are the inability to integrate in the host genome and the lack of unwanted genes such as toxins and antibiotic resistance determinants that could be transferred to its host through lysogenic conversion or recombination [47]. A genomic analysis of Vibrio phage Artemius showed that it was a lytic phage because no integrase or temperateness-related genes were identified. This was further corroborated by all the bioinformatic tools used such as INTEGRALL and Phage AI, which also indicated a lytic life cycle. Furthermore, no toxins or AMR-related genes were found, indicating that Vibrio phage Artemius could be considered to be safe for use in phage therapy applications. Gene-encoding predicted structural proteins were identified in the genome of the novel phage, including capsid protein, tail-length tape measure protein, tail tube protein, tail tubular protein and neck protein. An interesting gene found in the Vibrio phage Artemius genome was ORF29 encoding a PD-(D/E) XS nuclease. A search in the Conserved Domain Database (CDD) of NCBI revealed that this particular protein belongs to the superfamily of Cas4 nucleases. It has been suggested that phage-derived Cas4 leads to the acquisition of more host-derived spacers in type II-C CRISPR-Cas systems, enhancing the survival of the host and ultimately the bacteriophage whilst in a carrier state [48]. If this is the case, a further investigation is required in order to better understand the role of this particular gene, which will improve our knowledge of the dynamics of phage/host interactions.

High temperatures usually have a devastating effect on phage integrity, causing tail aggregation, detachment of the phage head and denaturation of the nucleic acid [49]. Interestingly, Vibrio phage Artemius withstood temperatures as high as 80 °C. It has been suggested that heat resistance may be the result of mutations or strong protein interactions [50] and it is usually a unique characteristic of the phage particle [51]. Although it is not common, phages that are resistant to high temperatures have been reported before [52], many of which were members of the of *Siphoviridae* family [53].

The host range of Vibrio phage Artemius was limited to strains of *Vibrio alginolyticus* as it was not able to infect the other congeneric species; thus, it was a species-specific phage. Broad host-range phages are considered to be ideal for phage therapy, especially in aquaculture where the diversity of the pathogenic strains and species is wide. Phages with a broad host range have been reported against *Vibrio alginolyticus* [14,41]. In terms of efficacy, Vibrio phage Artemius could efficiently prevent the growth of its host even at low MOIs, suggesting that it could be practically used in aquaculture where considerable quantities are required to treat large volumes of water. This was further corroborated by the large burst size of the novel phage.

In conclusion, Vibrio phage Artemius is a potential new species of the *Siphoviridae* family that infects *V. alginolyticus* and, based on its biological and genomic characteristics, could be considered for efficient and safe phage therapy applications.

## Figures and Tables

**Table 1:** Bacterial strains used in the host range assay (T: strain type; h: host). “Environmental” refers to an environmental isolation source whereas “clinical” refers to bacteria that were isolated from disease cases in various fish.

Strain	Species	Locality	Isolation Source
HCMR-2 Art1^h^	*V. alginolyticus*	Greece	Environmental
HCMR-1 Art1	*V. alginolyticus*	Greece	Environmental
HCMR-1 Art3	*V. alginolyticus*	Greece	Environmental
LAR41	*V. alginolyticus*	Greece	Environmental
LAR50	*V. alginolyticus*	Greece	Environmental
LAR73	*V. alginolyticus*	Greece	Environmental
LAR74	*V. alginolyticus*	Greece	Environmental
LAR76	*V. alginolyticus*	Greece	Environmental
Gal 019	*V. alginolyticus*	Greece	Environmental
LAR170	*V. alginolyticus*	Greece	Clinical
Gal 074	*V. alginolyticus*	Greece	Clinical
Gal 048	*V. alginolyticus*	Greece	Clinical
DSMZ2171	*V. alginolyticus*	Japan	Collection (T)
VhSerFrE	*V. harveyi*	Greece	Clinical
DSM 19623	*V. harveyi*	Massachusetts	Clinical (T)
VH2	*V. harveyi*	Greece	Clinical
VIB391	*V. campbellii*	Thailand	Clinical
LAR52	*V. tubiashii*	Greece	Environmental
MAN113	*V. splendidus*	Greece	Environmental
LAR194	*V. mediterranei*	Greece	Environmental

**Table 2:** Phages infecting *Vibrio alginolyticus* used for the phylogenetic analysis with a large terminase subunit.

Phage	Family	Accession Number	Isolation Country	Date of Isolation
Vb ValS X1	*Demerecviridae*	QKN88544.1	China	2020-06-15
Valm-yong1	*Myoviridae*	QGF21295.1	China	2020-03-09
Va2	*Myoviridae*	QPB08501.1	China	2020-11-15
phi-Grn1	*Myoviridae*	ALP46971	Greece	2016-03-01
phi-St2	*Myoviridae*	ALP47351	Greece	2016-03-01
V039C	*Myoviridae*	QIQ61300	China	2020-04-01
Vb ValS PJ32	*Siphoviridae*	QNJ59211.1	China	2020-08-30
Vb ValP VA-RY-4	*Siphoviridae*	UFD98283	China	2021-11-24
BUCT194	*Schitoviridae*	UAW01163	China	2021-09-22
USC-1	*Myoviridae*	QDH47503	Australia	2019-07-07
5 TSL-2019	*Myoviridae*	QCW23225	Australia	2019-07-10
NF	*Siphoviridae*	QGZ13228	China	2019-12-25
Vb ValP IME271	*Podoviridae*	ASR73874	China	2017-08-08
Vb ValP IME234	*Podoviridae*	QUE30183	China	2021-04-28

**Table 3:** Host range of the Artemius phage (h: host strain; +++: complete clearing; +: substantial turbidity throughout the cleared zone; −: no lysis).

Strain	Species	Lysis
HCMR-2 Art1^h^	*V. alginolyticus*	+++
HCMR-1 Art1	*V. alginolyticus*	+++
HCMR-1 Art3	*V. alginolyticus*	+++
LAR41	*V. alginolyticus*	+
LAR50	*V. alginolyticus*	+
LAR73	*V. alginolyticus*	+
LAR74	*V. alginolyticus*	+
LAR76	*V. alginolyticus*	+
Gal019	*V. alginolyticus*	+
LAR170	*V. alginolyticus*	+
Gal074	*V. alginolyticus*	+
Gal 048	*V. alginolyticus*	+
DSMZ2171	*V. alginolyticus*	+
VhSerFre	*V. harveyi*	−
DSM 19623	*V. harveyi*	−
VH2	*V. harveyi*	−
VIB391	*V. campbellii*	−
LAR52	*V. tubiashii*	−
MAN113	*V. splendidus*	−
LAR194	*V. mediterranei*	−

**Table 4:** Summary of Vibrio phage Artemius ORFs that were annotated with relevant information based on significant amino acid sequences and protein structural homologies (E-value ≤ 10^−3^).

	Predicted Function	Start	End	Length	Direction	NCBI BLAST Best Hit	E-Value
ORF1	Hypothetical protein	154	876	722	Forward	AUR84443.1	3.00 × 10^−99^
ORF2	Terminase small subunit	878	1279	401	Forward	AUR84444.1	2.00 × 10^−47^
ORF3	Hypothetical protein	1436	1786	350	Forward	no hit	
ORF4	Hypothetical protein	1773	2015	242	Forward	no hit	
ORF5	Terminase large subunit	2005	3813	1808	Forward	AUS01868.1	0
ORF6	Portal protein	3806	5053	1247	Forward	AUS01869.1	0
ORF7	Hypothetical protein	5031	5696	665	Forward	QZI87804.1	3.00 × 10^−81^
ORF8	Major capsid protein	5780	7135	1355	Forward	PIY67122.1	6.00 × 10^−155^
ORF9	Hypothetical protein	7222	7743	521	Forward	PIY67121.1	1.00 × 10^−17^
ORF10	Stopper protein	7733	8077	344	Forward	UNY40138.1	1.00 × 10^−11^
ORF11	Hypothetical protein	8070	8882	812	Forward	QZI86159.1	5.00 × 10^−80^
ORF12	Hypothetical protein	8883	9248	365	Forward	PIY67118.1	5.00 × 10^−10^
ORF13	Tail tube protein	9254	9952	698	Forward	UNY40117.1	2.00 × 10^−65^
ORF14	Hypothetical protein	9954	10,355	401	Forward	no hit	
ORF15	Hypothetical protein	10,460	10,582	122	Forward	no hit	
ORF16	Neck protein	10,582	11,103	521	Forward	AUS01881.1	1.00 × 10^−83^
ORF17	Tail-length tape measure protein	11,100	13,478	2378	Forward	AUR85334.1	3.00 × 10^−100^
ORF18	Hypothetical protein	13,478	13,975	497	Forward	QGF20993.1	4.00 × 10^−62^
ORF19	Hypothetical protein	13,972	14,487	515	Forward	UNY40123.1	4.00 × 10^−40^
ORF20	Hypothetical protein	14,484	14,825	341	Forward	QGF20994.1	9.00 × 10-^40^
ORF21	TMhelix-containing protein	14,813	17,599	2786	Forward	QGF21014.1	0.00 × 10^0^
ORF22	Tail tubular protein	17,596	20,157	2561	Forward	QGF21011.1	7.00 × 10^−23^
ORF23	Putative DNA polymerase	22,438	20,198	2240	Reverse	AUR81370.1	0.00 × 10^0^
ORF24	Hypothetical protein	23,663	22,494	1169	Reverse	AUR81371.1	2.00 × 10^−15^
ORF25	DNA primase	26,251	23,759	2492	Reverse	AUR81372.1	0.00 × 10^0^
ORF26	Hypothetical protein	26,506	26,261	245	Reverse	AUS01892.1	7.00 × 10^−4^
ORF27	Hypothetical protein	26,940	26,506	434	Reverse	AUR83587.1	2.00 × 10^−72^
ORF28	Hypothetical protein	28,094	26,946	1148	Reverse	AUR85121.1	5.00 × 10^−114^
ORF29	PD-(D/E) XS nuclease	29,235	28,099	1136	Reverse	AUS01895.1	8.00 × 10^−133^
ORF30	Hypothetical protein	30,173	29,241	932	Reverse	AUS01896.1	5.00 × 10^−34^
ORF31	Hypothetical protein	30,329	30,604	275	Forward	no hit	
ORF32	Hypothetical protein	30,601	30,867	266	Forward	no hit	
ORF33	Helicase	30,864	32,750	1886	Forward	AUR81379.1	0.00 × 10^0^
ORF34	Hypothetical protein	32,747	33,091	344	Forward	UAW01179.1	3.00 × 10^−16^
ORF35	N-acetylmuramoy-L-alanine amidase	33,281	33,742	461	Forward	AUS01901.1	5.50 × 10^−76^
ORF36	TMhelix-containing protein	33,747	33,923	176	Forward	AUS01902.1	1.00 × 10^−8^
ORF37	TMhelix-containing protein	33,928	34,230	302	Forward	AUS01903.1	6.00 × 10^−3^
ORF38	Hypothetical protein	34,256	34,387	131	Forward	AUS01904.1	5.00 × 10^−8^
ORF39	Hypothetical protein	34,723	34,409	314	Reverse	AUR81384.1	1.00 × 10^−14^
ORF40	Hypothetical protein	35,295	34,972	323	Reverse	AUS01906.1	2.00 × 10^−12^
ORF41	Hypothetical protein	35,692	35,276	416	Reverse	no hit	
ORF42	Hypothetical protein	36,462	35,695	767	Reverse	AUS01910.1	2.00 × 10^−56^
ORF43	Hypothetical protein	37,231	36,452	779	Reverse	no hit	
ORF44	Hypothetical protein	37,508	37,221	287	Reverse	no hit	
ORF45	Hypothetical protein	37,840	37,544	296	Reverse	no hit	
ORF46	Hypothetical protein	38,385	37,837	548	Reverse	no hit	
ORF47	Hypothetical protein	38,896	38,372	524	Reverse	AUR81476.1	6.00 × 10^−13^
ORF48	Hypothetical protein	39,322	38,915	407	Reverse	QZI87821.1	8.00 × 10^−35^
ORF49	Hypothetical protein	40,245	39,319	926	Reverse	AUR81389.1	1.00 × 10^−21^
ORF50	Hypothetical protein	40,627	40,884	257	Forward	no hit	
ORF51	HNH nuclease	40,877	41,305	428	Forward	AUR81392.1	6.00 × 10^−48^
ORF52	Hypothetical protein	41,766	41,969	203	Forward	QDP60675.1	1.00 × 10^−4^
ORF53	Hypothetical protein	41,977	42,300	323	Forward	QZI91906.1	6.00 × 10^−25^
ORF54	DNA-binding domain protein	42,297	42,458	161	Forward	AUR81395.1	6.00 × 10^−14^
ORF55	Hypothetical protein	42,461	42,838	377	Forward	YP_009103702.1	6.00 × 10^−27^
ORF56	Hypothetical protein	42,835	42,954	119	Forward	YP_007675934.1	8.00 × 10^−6^
ORF57	Hypothetical protein	42,967	43,173	206	Forward	WP_081230226.1	7.00 × 10^−26^

## Data Availability

The genome sequence of phage Vibrio phage Artemius is available in GenBank under accession number ON366409 and under Biosample and Bioproject accession numbers SAMN29862054 and PRJNA860748 respectively.
